# Automated Planning for Bias-Free Validation of Discrete Proton Arc Therapy for Oropharyngeal Cancer

**DOI:** 10.1016/j.ijpt.2025.101209

**Published:** 2025-10-29

**Authors:** Wens Kong, Merle Huiskes, Steven J.M. Habraken, Eleftheria Astreinidou, Coen R.N. Rasch, Ben J.M. Heijmen, Sebastiaan Breedveld

**Affiliations:** a Department of Radiotherapy, Erasmus MC Cancer Institute, Erasmus University Medical Center, Rotterdam, the Netherlands; b Department of Radiation Oncology, Leiden University Medical Center, Leiden, the Netherlands; c HollandPTC, Delft, the Netherlands

**Keywords:** Automated treatment planning, Beam angle optimization, Discrete proton arc therapy

## Abstract

**Purpose:**

To systematically compare fully automated planning for 36-field discrete proton arc therapy (36PAT) with: (1) intensity-modulated proton therapy (IMPT) with 4- and 6-field clinical beam-angle class solutions (4CS, 6CS), (2) IMPT with patient-specific, computer-optimized beam-angle configurations with 6, 8 or 10 fields (6BAO, 8BAO, 10BAO), and (3) 36-field equiangular ‘‘Utopia’’ IMPT (36Utopia; total focus on dosimetric quality, no delivery time considerations) for oropharyngeal cancer patients.

**Materials and Methods:**

All automated plan generations were performed with Erasmus-iCycle. An energy layer (EL) reduction algorithm was developed in Erasmus-iCycle to balance plan quality with delivery time in discrete PAT planning. Patient-tailored beam-angle configurations for BAO plans were obtained with the earlier published iCycle-pBAO. In 36Utopia plans, no EL reductions were applied. While beam configurations varied among approaches, all final plans were automatically generated using the published wish-list driven SISS-MCO optimizer with dosimetry-based sparsity-induced spot selection (SISS), followed by multi-criterial spot weight optimization (MCO) and resulting in Pareto-optimality in spot weights. The same wish-list for all delivery approaches prevented planning bias across approaches.

**Results:**

36PAT plans achieved organs at risk (OAR) doses and normal tissue complication probability (NTCP) approaching 36Utopia plans. Relative to CS plans, 36PAT plans reduced normal tissue dose, decreasing xerostomia and dysphagia NTCPs. Compared to 4CS, 36PAT reduced summed NTCPs for grade 2 toxicity by 6.1%-point (*P* = .002) and grade 3 by 2.1%-point (*P* = .002). For 6CS, reductions were 4.7%-point (*P* = .002) and 1.2%-point (*P* = .01), respectively. 36PAT plans also outperformed BAO plans with 6 and 8 fields but were comparable to 10BAO in OAR doses and NTCPs for similar EL numbers and Monitor Units (MU).

**Conclusion:**

PAT demonstrated superior dosimetric quality over clinical class solutions for oropharyngeal cancer and approached Utopia. Ten-field IMPT with personalized beam angles could be an alternative to 36-field PAT with similar expected toxicity, ELs and MUs, but a lower number of fields.

## Introduction

Proton arc therapy (PAT) is a planning and delivery technique of proton therapy that delivers conformal radiation doses to tumors via many beam directions, often by rotating the gantry and alternatively the patient.^1^ PAT has been reported to result in a lower integral dose,^2^, ^3^, ^4^, ^5^ higher conformality,^2^, ^3^, ^6^, ^7^, ^8^, ^9^, ^10^ and a superior linear energy transfer (LET) distribution in the target,^11^, ^12^, ^13^, ^14^, ^15^ and can be delivered without a range shifter and therefore utilizes a sharper penumbra.^16^

Two modes of delivery have been suggested for PAT: (1) dynamic delivery, where dose is continuously delivered while the gantry rotates around the patient, and (2) discrete delivery, where step-and-shoot delivery is used over a large number of control points. For dynamic delivery, treatment time reductions have been reported,^17^, ^18^, ^19^, ^20^, ^21^, ^22^ thereby allowing for increased patient throughput, but it also poses more challenges. It necessitates advanced technology for stable delivery; the system should deliver spots at submillimeter precision whilst rotating with precise synchronisation of spot delivery patterns with gantry motion. Furthermore, the gantry speed should be dynamically adjustable based on position feedback and treatment progress.^1^ These technological challenges may be even more pronounced for existing facilities as many of them operate with large and heavy gantries of 100–200 tons. Other technological limitations include long energy layer (EL) switching times in systems, especially for energy up-switching, and a lack of robust safety mechanisms for preventing gantry collisions during PAT delivery. Cyclotron- and synchrotron-based systems may require different approaches to clinical implementation of dynamic-PAT due to their different energy extraction methods, and corresponding energy switching times.

The treatment planning for dynamic PAT has been shown to be NP-hard.^23^ Although many beam angles may add degrees of freedom in terms of dosimetry, dynamic PAT planning also requires optimization of the total delivery time. The latter poses many complex restrictions compared to fixed-field intensity-modulated proton therapy (IMPT). Dynamic PAT treatment planning must consider machine-specific delivery characteristics such as gantry mechanical specifications, specifics of the EL selection and switching systems, spot irradiation sequences„ and scanning time. This can be performed through (greedy) heuristics,^2^, ^4^, ^7^, ^12^, ^13^, ^24^, ^25^, ^26^ surrogates^27^, ^28^, ^29^ or direct^30^ optimization. Optimal EL sequencing along the arc trajectory is needed to control delivery times. Often, this involves minimizing the number of EL switch-ups and reducing gantry braking.^22^ The final energy sequence depends on the type of accelerator and the delivery technique used (e.g., spot scanning, raster scanning, or line scanning). Delivery with a fixed beam line and rotating upright patient has been proposed as an alternative to gantry-based systems where the patient lies on a fixed couch,^31^, but many technical and treatment planning challenges valid for delivery with a rotating gantry still apply.

Discrete PAT, featuring dose delivery via many discrete beam directions while the beam is off in between beams, may provide an alternative solution to dynamic delivery, possibly at the expense of longer treatment times.^1^ To enable clinically acceptable delivery times with discrete PAT, the number of ELs per field (or control point) must be controlled.^32^ Discrete PAT using 360 ELs distributed in 30 directions showed a great dosimetric improvement compared to clinical 4-field IMPT in oropharyngeal cancer patients.^3^ Engwall et al.^33^ proposed a partitioning approach for discrete PAT to speed up delivery, in which discrete PAT plans are split into subplans with a lower number of control points to be delivered over different fractions in the treatment course. Improved target robustness was found in partitioned plans compared to IMPT, while orans at risk (OAR) doses and normal tissue complication probability (NTCP) values were almost as low as for plans for 3 oropharyngeal cancer patients.^33^

Fracchiolla et al*.*^10^ performed a retrospective comparison of clinically delivered IMPT plans with alternative discrete PAT plans for 7 sinonasal and 3 nasopharyngeal patients. They found significant reductions in PAT plans in the near maximum dose to the brainstem and a median NTCP reduction of 8.5% for xerostomia. Treatment times were similar. Based on a successful end-to-end commissioning they started to treat patients with their procedure for discrete PAT.

In this study, we used fully automated treatment planning to systematically compare for oropharyngeal cancer patients 36-field discrete proton arc therapy (36PAT) with: (1) IMPT with 4- and 6-field clinical beam-angle class solutions (4CS, 6CS), (2) IMPT with patient-specific, computer-optimized beam-angle configurations with 6, 8 or 10 fields (6BAO, 8BAO, 10BAO), and (3) 36-field equiangular ‘‘Utopia’’ IMPT plans (36Utopia); in contrast to 36PAT, no energy layer reduction was performed for 36Utopia, thereby aiming at the best possible dosimetry, without considering delivery time. For all 4 delivery approaches, plans were generated in a 2-step process, with the above described beam-angle pre-selection as the first step. In the second step, the final IMPT plan was generated based on the pre-selected angles (and patient-tailored energy layers in case of 36PAT), using fully automated planning with exactly the same optimization engine (Erasmus-iCycle),^34^ aiming at bias avoidance in treatment planning.

## Materials and methods

This study was set up and executed following the RATING guidelines for treatment planning studies^35^ and attained a score of 92% (RATING score sheet in the Supplementary Material).

### Patient cohort

Planning CT scans and delineations of 10 randomly selected oropharynx cancer patients, previously treated with IMPT on a Varian ProBeam system (Varian, a Siemens Healthineers Company), were used. The data originate from the research database of the Holland Proton Therapy Center. This database consists of data from all consenting patients treated at HollandPTC. The local Institutional Review Board waived the need to assess the protocol of the research database.

All patients were treated with a simultaneous integrated boost scheme, with 70 Gy_RBE_ prescribed to the primary tumor and positive lymph nodes (CTV70), and 54.25 Gy_RBE_ to bilateral elective node volumes (CTV54.25) in 35 fractions, in accordance with our clinical practice. The mean volumes of CTV70 and CTV54.24 were 83 mL (range: 27–187) and 270 mL (range: 153–353).

### IMPT treatment planning in Erasmus-iCycle

Details on beam-angle pre-selection for the 4 investigated delivery approaches (PAT, CS, BAO and Utopia) are described in Sections 2.3-2.5 below. For all approaches, the last step in plan generation was fully-automated wish-list driven multi-criterial generation of a final robust treatment plan,^34^ using the previously published SISS-MCO pipeline^36^ that is embedded in Erasmus-iCycle.

The applied automated multi-criterial plan generation in SISS-MCO was wish-list driven.^34^ In a wish-list, optimization rules are defined using hard constraints and prioritized objectives. Automated wish-list driven planning results for each patient in a single Pareto-optimal plan. Wish-lists are a priori generated for a patient population.^37^ Wish-list driven MCO avoids manual Pareto navigation, reliance on historical data as in machine learning, and knowledge based planning. The applied wish-list was the same for all the plans generated in this study for all 4 investigated delivery approaches, ensuring consistency and allowing bias-free comparisons. With this wish-list, SISS-MCO could generate plans in line with our clinical planning aims and trade-offs. In recent studies, SISS-MCO plans were reported to be superior to manually generated clinically delivered plans.^38^, ^39^

**Figure 1:**
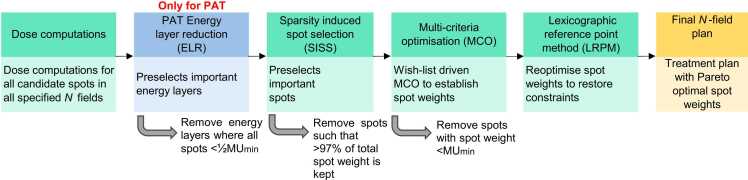
An overview of the optimization pipeline for final plan generation. All but the second block are in SISS-MCO, the second block is the additional phase is needed for PAT. See Appendix A for details on ELR. Abbreviations: ELR, energy layer reduction; MCO, multi-criteria optimization; PAT, proton arc therapy; SISS, sparsity-induced spot selection.

SISS-MCO has been described in detail in Kong et al*.*^36^ Here, a brief summary and diagram (Figure 1) is provided: Input for SISS-MCO were dose matrices for a large set of candidate pencil beams for predefined beam-angle directions and beam energies. SISS-MCO then created a robust treatment plan in 3 phases: (1) large-scale sparsity-induced spot selection (SISS), (2), scenario-based multi-criteria optimization (MCO) of MUs for the high-weight spots selected in phase 1, driven by the wish-list,^34^ and (3) removal of spots with MU below the clinically used minimum MU (MUmin), and restoration of constraints in the final plan with the Reference Point Method (Van Haveren et al.).^40^ Clinical minimum and maximum monitor unit constraints were always adhered to. By design, SISS-MCO plans are Pareto-optimal with regard to spot intensities. For an *N*-field IMPT plan, these phases constitute all but the second block of Figure 1.

### iCycle-PAT: Automated treatment planning for discrete proton arc therapy

A dedicated EL reduction (ELR) algorithm was developed for PAT in Erasmus-iCycle, aiming at high dosimetric plan quality with the total number of ELs in the 36 PAT fields as low as possible to control delivery time (second block in Figure 1). After EL selection, PAT plan optimization followed the same SISS-MCO planning phases as for the other investigated approaches (blocks 3, 4 and 5 in Figure 1).

ELR uses group regularization to sparsify the EL space for EL selection. The balance between the mean number of selected ELs in a patient population and the mean plan quality can be adjusted by the magnitude of the regularization coefficient the cost function. A full description of the ELR algorithm can be found in Appendix A.

### 4CS, 6CS and 36Utopia plans used for discrete PAT validation

For validation of 36PAT, we used SISS-MCO (Section 2.2) to automatically generate plans for our previous 4-field, and current 6-field clinical class solutions (4CS and 6CS). The following (gantry, couch) angles were involved in 4CS and 6CS: {(150^∘^, 0^∘^), (60^∘^, 0^∘^), (300^∘^, 0^∘^), (210^∘^, 0^∘^)} and {(200^∘^, 0^∘^), (260^∘^, 20^∘^), (310^∘^, 0^∘^), (50^∘^, 0^∘^), (100^∘^, 340^∘^), (160^∘^, 0^∘^)}. As in clinical practice, range shifters with 34 mm water equivalent thickness were used for all fields in the 4CS, and in all fields in the 6CS, except for the gantry angles 160^∘^ and 200^∘^.

For the creation of 36Utopia plans, the same candidate beams were used as for 36PAT, but the ELR step in Figure 1 was skipped, aiming at the highest dosimetric quality, without considering delivery times.

In this study, no comparisons with the original clinical plans were included. SISS-MCO plans were preferred to avoid planning bias. Comparisons between SISS-MCO- and clinical plans are presented in,^38^ showing superiority of SISS-MCO planning.

### Plans with patient-tailored beam-angle configurations to assess added value of discrete PAT

6BAO, 8BAO and 10BAO plans were generated with the previously published iCycle-pBAO approach that includes patient-specific automated BAO.^41^ In summary, iCycle-pBAO starts by solving a large-scale total-beam-space IMPT problem with 72 equiangular beams, using a cost function which is based on the wish-list, and includes a group normalization to sparsify the beam-angle space. Next, the 6, 8 or 10 beams with the highest weights were used for generation of the 6BAO, 8BAO and 10BAO plans with SISS-MCO (see^36^ and Section 2.2 for details of SISS-MCO).

### Treatment planning details

Minimum and maximum doses for both targets, and maximum doses for the brainstem, spinal cord, cochlea, optic nerves, optic chiasm, lenses, and mandible were optimized with scenario-based robust optimization^42^ in all delivery approaches, using the following 21 scenarios: the nominal scenario (1 scenario), proton range undershoot and overshoot scenarios of 3% in the absence of setup errors (2 scenarios), setup errors of 3 mm in positive and negative directions along 3 axes without range error (6 scenarios), and with undershoot (6 scenarios) and overshoot (6 scenarios) of 3%. Nominal scenario only mean dose minimization was used for the following OAR: parotids, submandibular glands, inferior/middle/superior PCM, larynx supraglottic, glottic area, esophagus, cricopharyngeus, brainstem, and spinal cord.

Consistent with clinical practice, for the 4 investigated planning approaches, spots traversing the maxillary sinuses, shoulders or metal dental fillings were upfront excluded. Maximum dose contributions per field were constrained to 47 Gy. All isocentres were positioned at the center of mass of CTV54.25.

### Use of range shifters

In current IMPT practice worldwide, most head and neck patients are treated with 3–6 IMPT fields. In most cases, range shifters are used in all or most fields to adequately treat superficial parts of the target. Range shifter use in the investigated 4-field and 6-field CS is described in Section 2.4.

A disadvantage of using range shifters is that the lateral beam penumbra widens, which may result in increased OAR doses. In this study, we hypothesized that with larger numbers of treatment fields, range shifters may not always be needed. To investigate this, for 36PAT, 36Utopia and 6/8/10BAO, 2 plans per patient were generated, one without and the other with range shifters with 34 mm water equivalent thickness in all fields.

### Plan evaluations and comparisons

All generated plans were normalized such that CTV70 D98% in the 21-scenario voxel-wise minimum dose distribution (VWmin)^43^ was equal to 95% of the prescription dose, comparable to clinical practice. NTCPs for grade 2 (G2) and grade 3 (G3) xerostomia and dysphagia were determined according to the Dutch National Protocol for Model-Based Selection for Proton Therapy in Head and Neck Cancer.^44^

The reported D_mean_ were calculated in the nominal scenarios and the near-maximum doses were derived from the voxel-wise maximum (VWmax) dose distributions in accordance with clinical practice. Differences between planning approaches were tested for significance (*P* < .05) using 2-sided Wilcoxon signed-rank tests.

## Results

All treatment plans for all patients across the 4 planning approaches met all institutional target and OAR dose constraints.

### Energy layer reduction (ELR) in iCycle-PAT

Prior to generating PAT plans, investigation was performed on the regularization coefficient for ELR in iCycle-PAT to explore the balance between the number of final ELs in 36PAT plans and the resulting plan quality. A population average of 394 ELs was chosen as the best trade-off between number of ELs and obtained NTCPs. A full analysis is included in Appendix B. Energy layer selection took on average 90 minutes per patient (range: 64–140).

### Validation of iCycle-PAT: 36PAT vs. 4CS, 6CS and 36Utopia

36PAT and 36Utopia plans without range shifters showed better dosimetry than their range shifter counterparts. The former plans were therefore used in this section.

**Figure 2:**
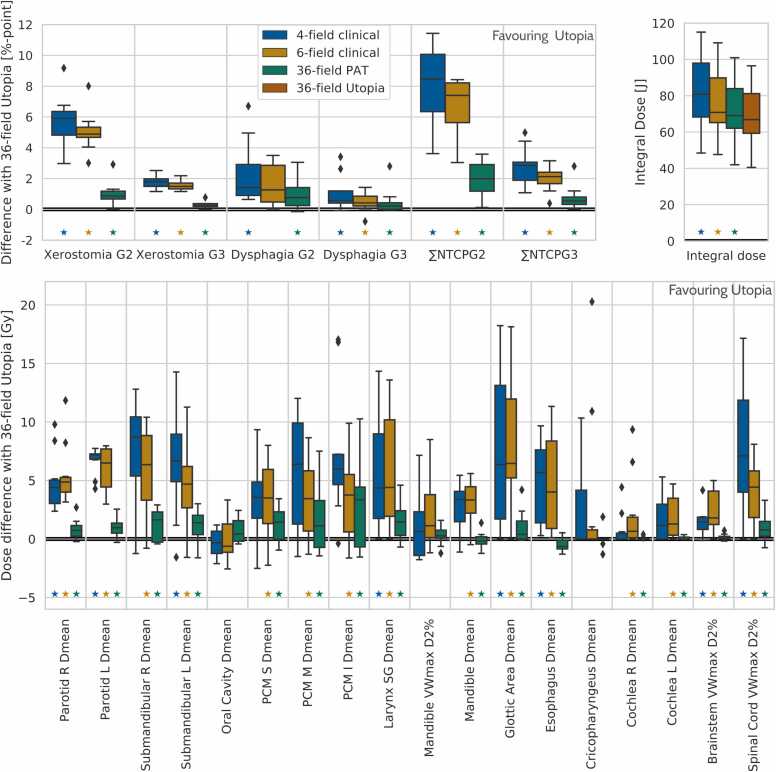
Comparisons of NTCPs and integral doses (upper panels) and OAR dose metrics (lower panel) for 36PAT and 4CS and 6CS with 36Utopia. Significance (*P* < .05) is indicated by asterisks. Horizontal bars indicate median values. Whiskers include all differences except for outliers, defined using the interquartile range (IQR) as: outside Q1–1.5 ⋅ IQR and Q3 + 1.5 ⋅ IQR.

**Figure 3:**
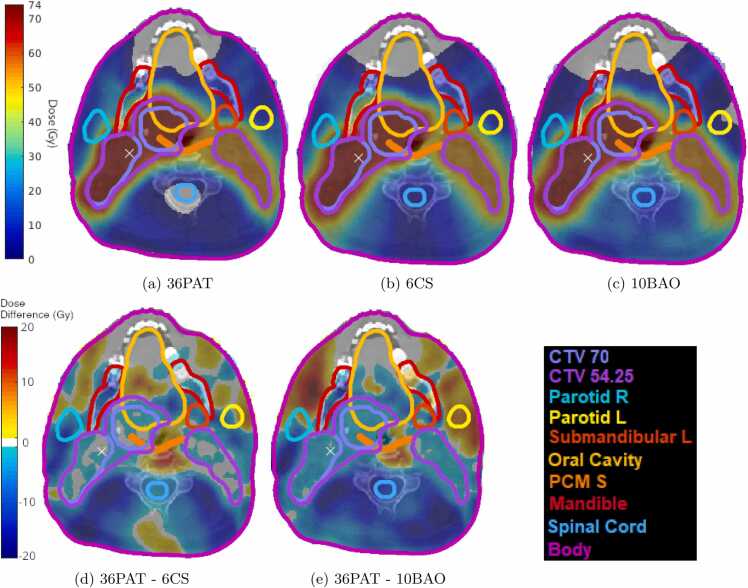
Dose distributions for: (a) 36PAT, (b) 6CS and (c) 10BAO in an axial plane for an example patient, and the corresponding dose differences for (d) 36PAT - 6CS and (e) 36PAT - 10BAO. Positive and negative dose differences correspond to a higher and lower dose in 36PAT respectively.

Figure 2 compares NTCPs, integral doses and OAR dose metrics for 36PAT and 4CS and 6CS, while using 36Utopia as benchmark. While 36PAT plans had on average 394 ELs, 36Utopia plans had on average 987 ELs. Nonetheless, 36PAT approached 36Utopia for all NTCPs, OAR dose metrics and integral doses, showing only a small dosimetric impact of the substantial reduction in ELs. Compared to 4CS and 6CS, 36PAT showed significant superiority in all aspects. A comparison of 36PAT and 6CS dose distributions is shown in Figure 3 for an example patient.

### Added value of 36PAT compared to 6BAO, 8BAO and 10BAO

For 6BAO, all dosimetric parameters favored range shifter insertion, where this was the case in 6/10 plans for 8BAO and 3/10 plans for 10BAO (see Appendix C).

**Figure 4:**
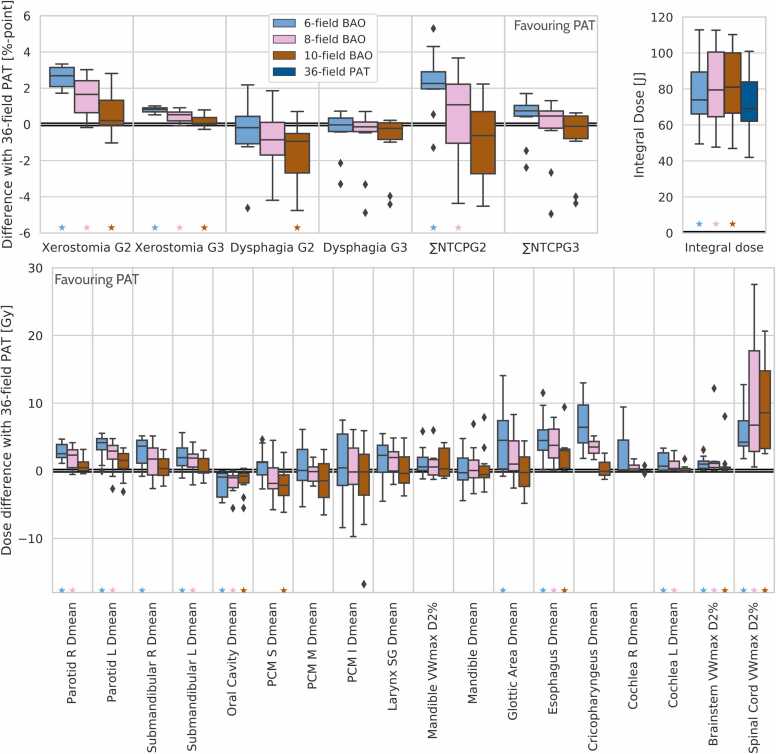
Comparisons of NTCPs and integral doses (upper panels) and OAR dose metrics (lower panel) for 6-, 8- and 10BAO with 36PAT. Significance (*P* < .05) is indicated by asterisks. Horizontal bars indicate median values. Whiskers include all differences except for outliers, defined as: outside Q1–1.5 ⋅ IQR and Q3 + 1.5 ⋅ IQR.

Differences between 36PAT and 6/8/10BAO in NTCPs, integral doses and OAR dose metrics are shown in Figure 4. 36PAT outperformed 6BAO and 8BAO, whereas 10BAO and 36PAT had overall similar quality for NTCPs and OAR dose metrics with some pros and cons for both, while the latter clearly showed favorable integral doses. Apart from fewer beams (10 vs. 36), 10BAO also used slightly less ELs (389 vs. 394) and spots (2527 vs. 2737). Plan optimization times were slightly shorter for 36PAT compared to 10BAO: 330 minutes (253−535) vs. 387 minutes (212−537).

**Figure 5:**
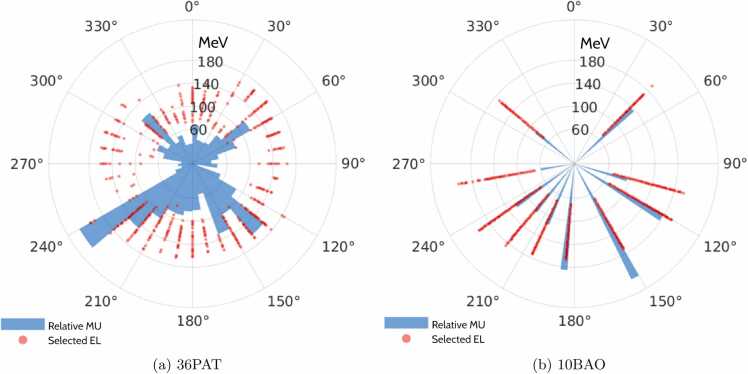
Selected energy layers (ELs) (MeV) and relative monitor units (MUs) per field for patient 1 for (a) 36PAT and (b) 10BAO.

For an example patient, Figure 5 shows the angular EL and relative monitor units distributions.

## Discussion

In this work, we used fully automated treatment planning to systematically compare for oropharyngeal cancer patients 36-field discrete proton arc therapy (36PAT) with: (1) IMPT with 4- and 6-field clinical beam-angle class solutions (4CS, 6CS), (2) IMPT with patient-specific, computer-optimized beam-angle configurations with 6, 8 or 10 fields (6BAO, 8BAO, 10BAO), and (3) 36-field equiangular ‘‘Utopia’’ IMPT plans (36Utopia). In contrast to 36PAT, no energy layer reduction was performed for 36Utopia, thereby aiming at the best possible dosimetry, without considering delivery time. While beam angles were different for the 4 investigated delivery approaches, final plan generations based on pre-selected angles were all performed automatically with Erasmus-iCycle with the same wish-list, aiming at planning bias avoidance. Erasmus-iCycle^34^ was configured for generating plans in line with our clinical aims and trade-offs.^39^ To the best of our knowledge, this is the first treatment planning study that uses a single optimizer for automated generation of treatment plans for a broad range of proton delivery approaches, including PAT.

36-field PAT showed dosimetrically superior to clinical beam-angle class solution IMPT plans with 4 and 6 fields, while closely approaching 36-field Utopia plans, the latter with 2.5 times more energy layers than 36-field PAT. 36-field PAT was also dosimetrically superior to 6-field and 8-field IMPT with patient-tailored beam-angle configurations. On the other hand, 36-field PAT was overall similar to 10-field IMPT with patient-tailored beam-angle configurations regarding NTCPs and OAR doses. Near-maximum spinal cord doses were higher for 10-field IMPT, but always well within constraints. Integral doses were low, and lowest for 36-field PAT. The number of energy layers and Monitor Units for 36PAT and 10BAO were similar. Therefore, 10-field IMPT with personalized beam angles could be an alternative to discrete 36-field PAT in oropharyngeal cancer patients with similar expected toxicity, number of energy layers and Monitor Units, but at a much lower number of fields. Important to realize is that our study investigated oropharyngeal cancer patients, treated in line with our clinical protocol. More research is needed for other protocols and tumor sites for drawing more broad conclusions.

We found that for all involved patients, 36PAT plan quality was clearly highest without the use of range shifters (data not presented). This is likely because the large number of fields could always guarantee appropriate coverage of superficial target areas, allowing the benefit of smaller lateral penumbrae in the absence of range shifters. For 10BAO, not for all patients, the use of range shifters could be omitted (Appendix C). In this study, we addressed the issue by generating two 10BAO plans per patient, one with and the other without range shifters in all beams, and then selected the best plan for each patient. This solution is suboptimal because it necessitates the generation of 2 plans, although this plan generation is fully automatic. Moreover, we only considered plans with range shifters in all fields, or no range shifters at all. For certain patients, the optimal IMPT plan may involve using a range shifter in selected fields while omitting it in others. Integrating this selection procedure in automated planning could possibly further improve the quality of 10BAO plans compared to 36PAT. However, this approach may increase the complexity of quality assurance and treatment delivery and could disrupt workflow, particularly when treatments are delivered from many directions. Practical implementation of per-field range shifter optimization is currently challenging, as exhaustive searches including all combinatorial options would take prohibitively long calculation times. A heuristic for including this in the optimizations would need to be developed.

For CS, BAO and 36Utopia, the same EL separation was used for plan generations; for 36PAT this separation was used as a starting point with ELR being used to reduce the number of ELs and thereby the mean separation between them. Increasing the EL resolution could potentially result in further enhanced plan quality, but it could also increase the number of ELs and thereby treatment time. Future research is needed to explore this aspect.

In this study, we investigated discrete PAT with 36 equiangular fields. In principle, this could have been any number, but limitations in computational resources prohibited using larger field numbers. The capacity of the available hardware with 80 GB of GPU memory being the largest available on the market at the time of writing limited the further increase in number of candidate spots. PAT optimization needed a sufficient number of spots per energy layer for the energy layer preselection. Therefore, more fields (e.g. also non-coplanar) were not considered. The impact of adding more coplanar fields on plan quality could be minor given the similarity in plan quality of 36PAT and 10BAO. With the pipeline depicted in Figure 1, subsequent plan optimization after ELR took about 4 hours, with SISS in tens of minutes and LRPM under 5 minutes.^36^ Increasing the number of fields in PAT, and thereby candidate energy layers, is expected to increase the ELR optimization time. Plan optimization times for subsequent optimization phases are less influenced by the number of initial ELs as many spots are discarded after energy layer reduction.

Compared to De Jong et al*.*^3^, our discrete PAT showed slightly less NTCP gain compared to 4CS plans. This could be due to the comparison with high-quality automatically generated 4CS plans in our study, compared to comparisons with clinical plans by De Jong et al.^3^. We used automated planning with SISS-MCO for generation of the 4CS plans. In recent studies, we demonstrated the superiority of such plans over clinical (manual) planning.^38^, ^39^ Another factor might be that blocking pencil beams through maxillary sinuses, shoulders and metal dental fillings was applied for both PAT and clinical template plans in this study, whereas in De Jong et al.^3^ this was applied only in manual plans. Blocking these structures limits the degrees of freedom available to the optimizer.

Fracchiolla et al.^10^ reported larger NTCP reductions with discrete 30-field PAT vs clinical 6-field IMPT than observed in this study. This could be due to differences in investigated tumor sites, i.e. sinonasal and nasopharynx by Fracchiolla et al. vs. oropharynx in this study. Possibly, also the use of manual planning vs. automated planning, the latter used in this study, could have influenced the observations.

Engwall et al.^33^ proposed 30-field PAT partitioned into subplans with 10 and fields to be delivered every third (3 × 10) or 7th fraction (7 × 10). Partitioning improves daily delivery time but may add complexity to treatment planning and possible plan adaptations. They also observed that the partitioning of the PAT plans reduced the OAR sparing benefits of PAT, as in every fraction, the minimum and maximum target dose constraints were imposed. Furthermore, biologically equivalent OAR doses must be accounted for when distributing doses differently over different fractions.^33^ Future research should include dosimetric and practicality comparisons between 10BAO and interlaced 10-field PAT subplans.

Radiation-induced lymphopenia is mentioned in discussions on advantages and disadvantages of PAT vs. strategically placed fewer fields. Since radiation-induced lymphopnia is, at least in some cases, correlated with treatment outcomes,^45^ the spatial and temporal distribution of low and mid-level doses is important. Longer treatment times and interruptions in delivery influence the lymphocyte population, as larger volumes of blood are exposed.^46^, ^47^ Either strategy warrants research on lymphocyte counts. As others, we observed low integral doses for all investigated delivery approaches compared to those reported for photon therapy (VMAT), and that 36PAT had lower integral doses than strategies with fewer beams, which could be favorable to reduce risk of lymphopenia. In this study, we did not include dose minimization in structures related to lymphopenia.

As briefly described in the Introduction section, dynamic PAT has the potential for fast dose delivery and promising dosimetric quality compared to few-field IMPT, but technological advances are needed prior to clinical introduction.^1^ On the other hand, high-quality discrete PAT has been introduced clinically using existing technology.^10^ To our knowledge, there are no comprehensive studies that have compared dynamic PAT, discrete PAT and beam-angle optimized IMPT using limited numbers of fields (4−10), considering both plan quality and delivery time. Our study pointed at the importance of including also 10-field IMPT plans in this type of comparative studies, because of similar plan quality as 36PAT for oropharyngeal tumors, with much fewer fields.

From the literature, it is known that the delivery time of a discrete PAT treatment may be greatly dependent on the additional time needed between fields for manual interactions with the treatment software and machine, and security checks.^33^, ^3^, ^16^ Engwall et al.^33^ reported that the delivery time for 30 beam directions being continuously delivered just takes over 10 minutes. However, when adding time between fields, the total delivery time increases to around 15, 20, and 25 minutes with delays of 10, 20, and 30 seconds per beam direction respectively. This was around 5 to 8 minutes for 4/5-field IMPT. This is in line with the findings of Fracchiolla et al.,^10^ who reported 36 and 25 minutes for PAT with 30 and 20 beam directions respectively. The latter reported a similar delivery time (31 minutes) for 6 beams noncoplanar IMPT.^10^ This long delivery time could be caused by partial range shifter usage in 5 beam directions, which made the total number of fields 11, more than the 6 fields in our clinical solution.

Robustness against anatomical changes of dynamic, discrete and 10-field IMPT should be compared in future studies. PAT plans could be more sensitive as a consequence of improved high dose conformality. Especially in the head and neck area, where anatomical changes between treatment fractions are expected. The benefit of reaching high dose conformality might translate into an increased need for replanning or plan adaptation during the treatment course.^10^

This is the first PAT study to include a Utopia planning strategy in the comparisons. Utopia plans have the same number of fields as PAT plans, but are designed for the best possible dosimetry in coplanar treatments, without consideration of delivery time. We believe that Utopia plans can be considered as plan-quality benchmark for other planning strategies with more realistic delivery times. We demonstrated that our 36PAT- and 10BAO plans closely approached the quality of 36Utopia.

## Conclusion

Using a single optimizer for automated generation of all treatment plans, 36-field proton arc therapy plans were compared with 3 alternative IMPT strategies. For patients with oropharyngeal cancer, 36-field proton arc therapy showed superior dosimetric quality compared to clinical 4- and 6-beam-angle class solution plans, and very closely approached the quality of 36-field Utopia plans, the latter generated with maximum focus on dosimetry and no considerations for treatment time. Similarly, 10-field IMPT plans with personalized, computer-optimized beam angles also approached the dosimetric quality of 36-field Utopia.

## CRediT authorship contribution statement

**Wens Kong:** Conceptualization, Formal analysis, Investigation, Visualization, Writing – original draft, Writing – review & editing. **Merle Huiskes:** Data curation, Methodology, Writing – review & editing. **Steven J.M. Habraken:** Methodology, Writing – review & editing. **Eleftheria Astreinidou:** Writing – review & editing. **Coen R.N. Rasch:** Funding acquisition, Writing – review & editing. **Ben J.M. Heijmen:** Conceptualization, Funding acquisition, Methodology, Supervision, Writing – original draft, Writing – review & editing. **Sebastiaan Breedveld:** Conceptualization, Data curation, Investigation, Methodology, Supervision, Writing – original draft, Writing – review & editing.

## Declaration of Competing Interests

The authors declare the following financial interests/personal relationships which may be considered as potential competing interests: Wens Kong reports financial support was provided by Varian Medical Systems Inc. If there are other authors, they declare that they have no known competing financial interests or personal relationships that could have appeared to influence the work reported in this paper.

## Appendix A Supplementary material

Supplementary materialSupplementary material

## Footnotes

Appendix A Supplementary data associated with this article can be found in the online version at doi:10.1016/j.ijpt.2025.101209.
