# Supplementary material for: Automated Planning for Bias-Free Validation of Discrete Proton Arc Therapy for Oropharyngeal Cancer
Source: Int J Part Ther. 2025 Oct 29;18:101209. doi: 10.1016/j.ijpt.2025.101209 (PMC12746094; doi:10.1016/j.ijpt.2025.101209)
Supplement: Supplementary file 1 — Supplementary materialSupplementary material [file mmc1.pdf]

Appendices

A Energy Layer Reduction for iCycle-PAT

Energy Layer Reduction (ELR) employed for each patient a large surrogate optimisation problem with 120,000 candidate spots. This number was chosen to match the capacity of the GPU used (details on computer hardware used below). All spots were positioned within a volume comprising the composite of the CTVs in all 21 robustness scenarios, enlarged by 5 mm. Within this volume, candidate ELs for each beam were automatically selected based on the volume and the pre-defined EL spacing (3.5 mm). For each patient, spots were laterally sampled uniformly in all ELs. The dose deposition matrices for all spots were calculated for all structures using the ASTROID dose engine [1].

The applied weighted-sum cost function for ELR (Equation 1) closely resembles the function used for spot selection in SISS [2], with the primary distinction being the addition of the group sparsity regularisation norm ($L_{1,2}$-norm) $||\boldsymbol{x}||_{1,2}$, which was incorporated to sparsify energy layers. Quadratic functions $Q_i(\boldsymbol{x})$ in Equation 1 were used for all target underdose and overdose objectives, while maximum and mean dose objectives $\Lambda_j(\boldsymbol{x})$ were applied for the OARs. $Q_i(\boldsymbol{x})$ and $\Lambda_j(\boldsymbol{x})$ reflect the dosimetric aims in the wish-list. The vector norm (L1) $||\boldsymbol{x}||_1$ aims at sparsifying spots (like in SISS, Kong *et al.* [2]).

After obtaining a solution for the surrogate problem (MUs for all spots), energy layers with spot weights $\leq \frac{1}{2}\mathrm{MU}_{\mathrm{min}}$. were removed. The remaining layers were used for the generation of the final plan with SISS-MCO . The MU-threshold was chosen based on investigations on a test set that was not included in the cohort of this study.

$$\underset{\boldsymbol{x}}{\text{minimise}} \quad \sum_{i=1}^{N_Q} \mathrm{Q}_i(\boldsymbol{x}) + \sum_{j=1}^{N_\Lambda} w_j \Lambda_j(\boldsymbol{x}) + \lambda_1 ||\boldsymbol{x}||_1 + \lambda_2 ||\boldsymbol{x}||_{1,2} \tag{1}$$

$$\text{subject to} \quad \boldsymbol{x} \geq 0 \tag{2}$$

where

$$\begin{aligned} \boldsymbol{x} &= \text{spot intensity vector} \\ N_Q &= \text{number of quadratic objectives} \\ N_\Lambda &= \text{number of linear objectives} \\ \mathrm{Q}_i &= \text{quadratic objectives (Equation 3) for targets and target rings} \\ \Lambda_j &= \text{linear OAR objectives (Equation 4)} \\ w_j &= \text{weighting coefficients for the linear OAR objectives (Equation 5)} \\ \lambda_1, \lambda_2 &= \text{coefficient for the vector and group sparsity norms} \end{aligned}$$

and with target objective functions, $\mathrm{Q}_i(\boldsymbol{x})$, defined by

$$\mathrm{Q}_i(\boldsymbol{x}) = \frac{1}{N_i}\mathrm{Q}_i(\boldsymbol{A}_i, \boldsymbol{x}, D_{\mathrm{ref}}, M) = \frac{1}{N_i}\sum_{k=1}^{N_i} \max\big(0, M \cdot (\boldsymbol{A}_{i;k}\boldsymbol{x} - D_{\mathrm{ref}})\big)^2 \tag{3}$$

where

$$N_i = \text{number of voxels in structure } i$$
$$D_{\text{ref}} = \text{reference dose value in accordance with the wish-list}$$
$$\boldsymbol{A}_i = \text{dose deposition matrix for quadratic objectives } i$$
$$\boldsymbol{A}_{i;k} = \text{row } k \text{ of dose deposition matrix } \boldsymbol{A}_i$$
$$\text{sgn}(M) = -1 \text{ for underdose penalty, 1 for overdose penalty}$$
$$|M| = 10000 \text{ for constraints, 50 for target dose objectives}$$

and with OAR objectives, $\Lambda_j(\boldsymbol{x})$, with weights, w_j, defined by

$$\Lambda_j(\boldsymbol{x}) = \Lambda_j(\boldsymbol{A_j}, \boldsymbol{x}) = \begin{cases} \max(\boldsymbol{A_j x}), & \text{minimise maximum OAR dose} \\ \text{mean}(\boldsymbol{A_j x}), & \text{minimise mean OAR dose} \end{cases} \tag{4}$$

$$w_j = \frac{1/\sqrt{p_j}}{||\boldsymbol{p}^{-\frac{1}{2}}||_1} \tag{5}$$

where

$$\boldsymbol{A_j} = \text{dose deposition matrix for linear objectives } j$$
$$\boldsymbol{p} = \text{wish-list priorities of linear objectives}$$
$$p_j = \text{wish-list priority of linear OAR objective } j$$

The weight λ_1 in Equation 1 was chosen the same as for SISS (10^{-4}), while λ_2 was defined through a grid search over a specified grid with equispaced values, these were $\{10^n \mid n \in \{-4, -3, \ldots 3\}\}$. The magnitude of λ_2 affects the number of selected ELs, but as a consequence also the degrees of freedom to the optimiser in plan generation. For final λ_2 selection, number of ELs was confronted with plan quality.

The maximum MU-constraint was not considered in ELR, but was accounted for in the subsequent SISS to enhance optimiser-convergence. Clinical MU bounds were enforced in consecutive phases.

The surrogate problem defined by Equations 1 and 2 was solved using the L-BFGS-B [3–5] solver until machine floating-point precision convergence. The optimisations were performed on a computer node equipped with an Intel Xeon Gold 6248R @3.00 GHz CPU and an NVIDIA A100 GPU.

References

[1] H. M. Kooy *et al.*, "A case study in proton pencil-beam scanning delivery," *International Journal of Radiation Oncology* Biology* Physics*, vol. 76, no. 2, pp. 624–630, 2010.

[2] W. Kong *et al.*, "Siss-mco: Large scale sparsity-induced spot selection for fast and fully-automated robust multi-criteria optimisation of proton plans," *Physics in Medicine and Biology*, 2024a.

[3] R. H. Byrd, P. Lu, J. Nocedal, and C. Zhu, "A limited memory algorithm for bound constrained optimization," *SIAM Journal on scientific computing*, vol. 16, no. 5, pp. 1190–1208, 1995.

[4] C. Zhu, R. H. Byrd, P. Lu, and J. Nocedal, "Algorithm 778: L-bfgs-b: Fortran subroutines for large-scale bound-constrained optimization," *ACM Transactions on mathematical software (TOMS)*, vol. 23, no. 4, pp. 550–560, 1997.

[5] J. L. Morales and J. Nocedal, "Remark on "algorithm 778: L-bfgs-b: Fortran subroutines for large-scale bound constrained optimization"," *ACM Transactions on Mathematical Software (TOMS)*, vol. 38, no. 1, pp. 1–4, 2011.

B Results of PAT energy layer reduction (ELR) tuning

The candidate spot set for 36PAT plan generations comprised on average 1575 ELs (range: 1312-1968) for the 10 study patients. A grid search (tuning) was performed to establish a suitable coefficient λ_2 for the $L_{1,2}$-norm in Equation 1, which affects the number of selected energy layers to be used for 36PAT. With larger values of λ_2 the number of selected ELs reduced, but as a consequence also plan quality in terms of the normal tissue complication probability (NTCP) for xerostomia and dysphagia and target coverage declined. As depicted in Figure 1, the average number of selected ELs varied from 139 for $\lambda_2 = 10^3$ to 830 for $\lambda_2 = 10^{-4}$.

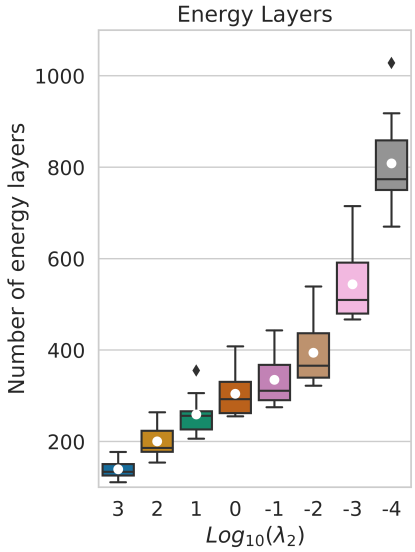

Figure 1: The resulting number of energy layers (ELs) in 36-field PAT for all 10 study patients, for all investigated λ_2. The black horizontal bars indicate median values, the white dots indicate the mean values. Whiskers include all differences except for outliers, defined as: outside Q1–1.5 $\cdot$ IQR and Q3 + 1.5 $\cdot$ IQR.

For λ_2 equal to 10^3, 10^2, and 10^1, the resulting numbers of energy layers were insufficient to ensure adequate target coverage in 6, 4, and 1 patients respectively. These λ_2 values could therefore already for this reason be excluded. Figure 2 shows for each mean number of ELs resulting from an investigated λ_2 value, the NTCPs for G2 and G3 xerostomia and dysphagia and their summations (ΣNTCPG2 and ΣNTCPG3), and, the integral doses. Figure 3 shows OAR doses as a function of the mean number of ELs. NTCPs, integral doses and OAR doses improved for increasing numbers of ELs (decreasing λ_2), but this effect plateaued at $\lambda_2 = 10^{-3}$ with 545 ELs on average. $\lambda_2 = 10^{-2}$ resulted in slightly higher NTCPs and integral doses, but since the number of ELs was substantially smaller (394). Therefore, presented 36PAT plans were generated using $\lambda_2 = 10^{-2}$.

Figure 4 shows angular EL distributions for an example patient.

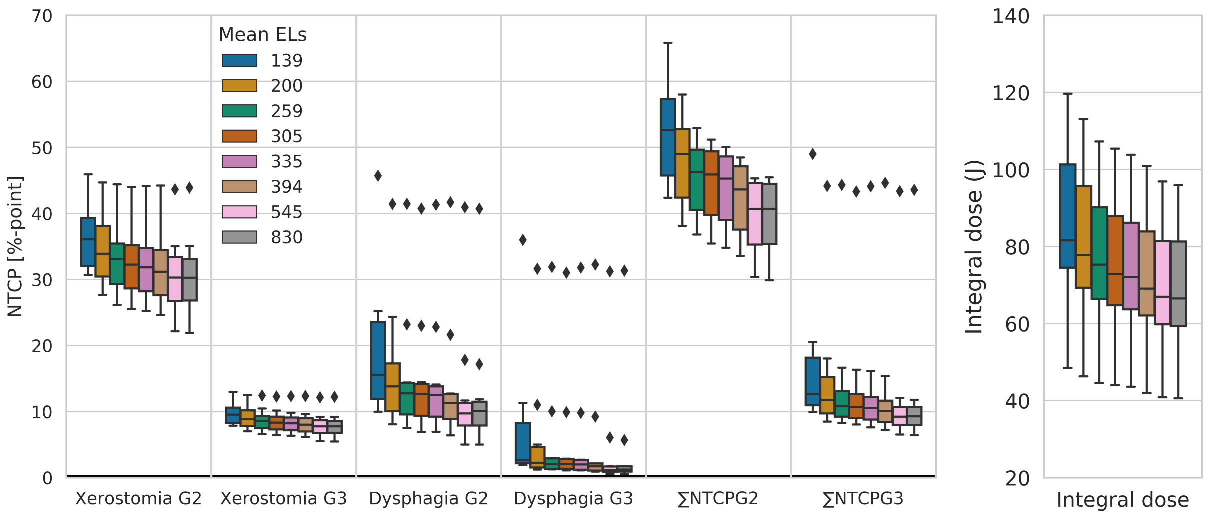

Figure 2: NTCPs (left) and integral doses (right) for each mean number of energy layers (ELs) resulting from investigated λ_2 (Figure 1) in 36-field PAT. Horizontal bars indicate median values. Whiskers include all differences except for outliers, defined as: outside Q1–1.5 $\cdot$ IQR and Q3 + 1.5 $\cdot$ IQR. For ELs = 139, 200 and 259, some patients did not have required target coverage.

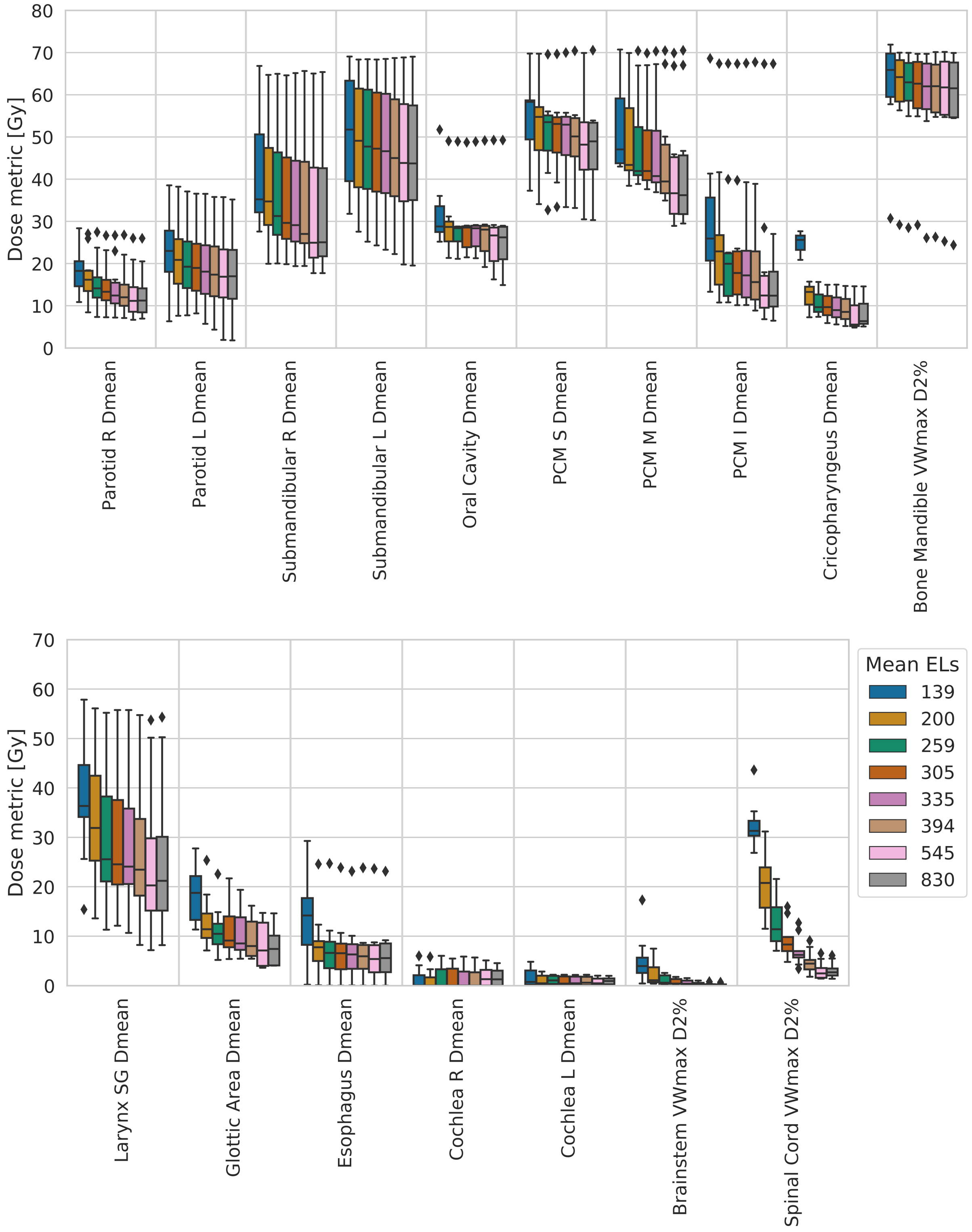

Figure 3: OAR dose metrics for each mean number of energy layers (ELs) resulting from investigated λ_2 (Figure 1). Horizontal bars indicate median values. Whiskers include all differences except for outliers, defined as: outside Q1–1.5 · IQR and Q3 + 1.5 · IQR. For ELs =139, 200 and 259, some patients did not have required target coverage.

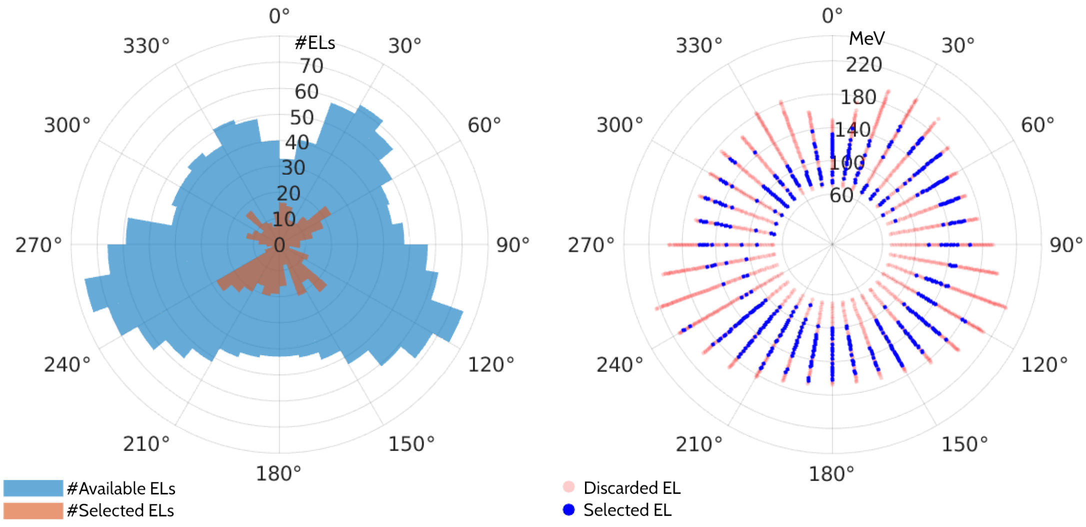

(a) (b)

Figure 4: Angular distributions showing energy selection for 36PAT for patient 1, (a) available and selected numbers of ELs, and (b) selected and discarded ELs.

C Rangeshifter usage

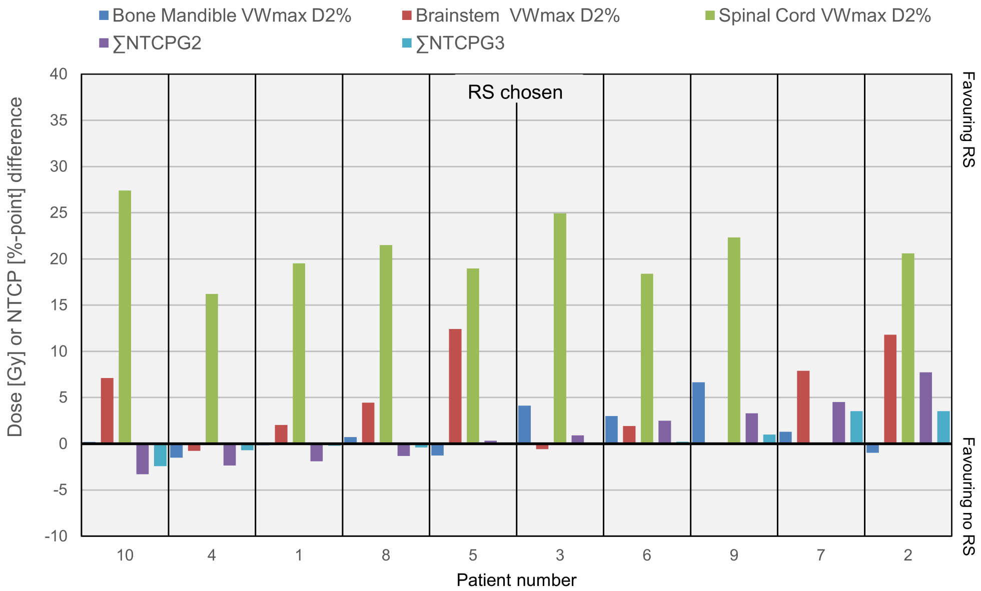

Figure 5: Serial OAR VWmax D2% and summed NTCP differences for 6-field with and without range shifter (RS).

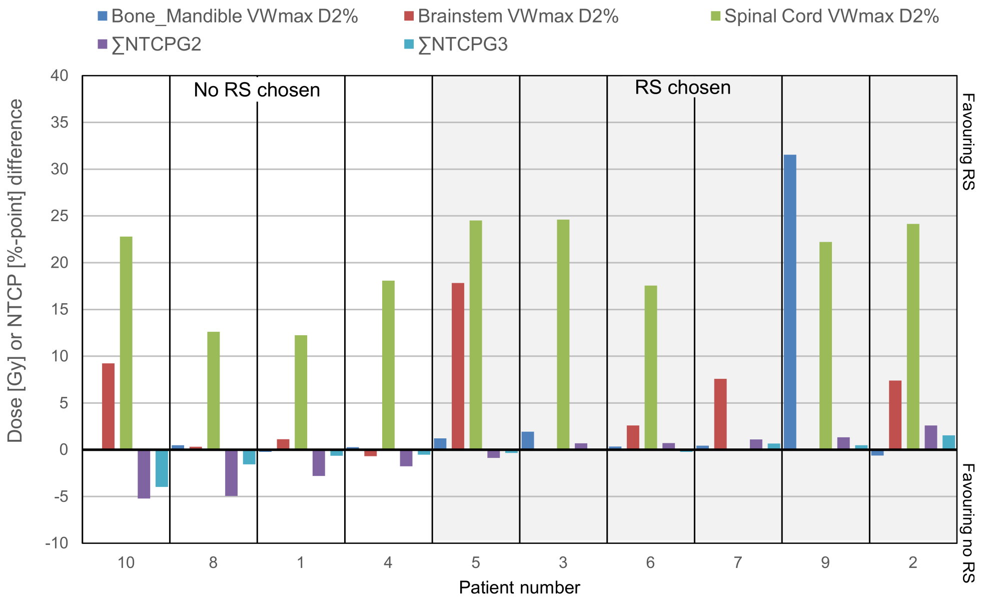

Figure 6: Serial OAR VWmax D2% and summed NTCP differences for 8-field with and without range shifter (RS).

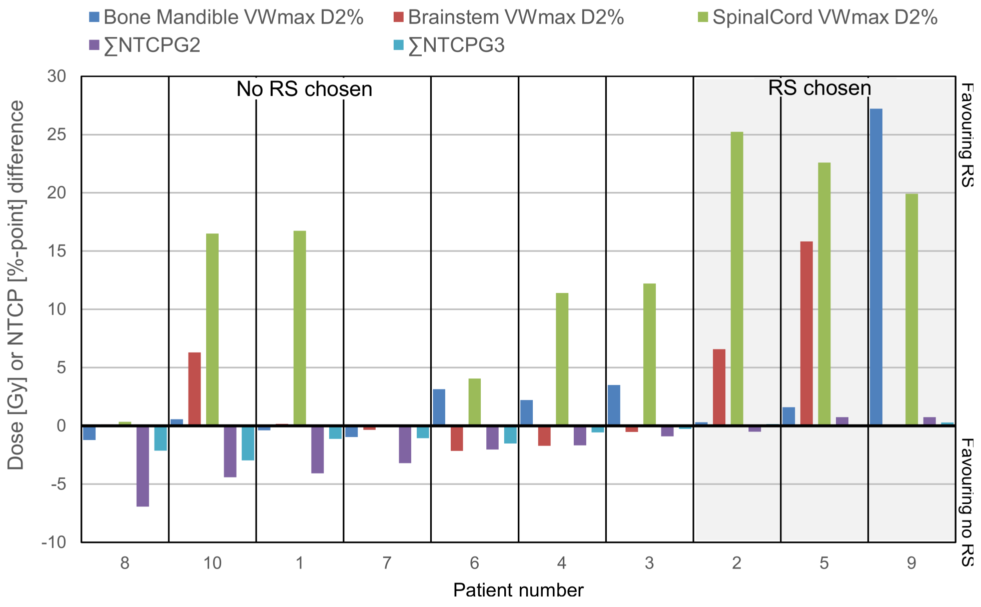

Figure 7: Serial OAR VWmMax D2% and summed NTCP differences for 10-field with and without range shifter (RS).
